# Activation of the G-protein coupled receptor GPR35 by human milk oligosaccharides through different pathways

**DOI:** 10.1038/s41598-020-73008-0

**Published:** 2020-09-30

**Authors:** Francis Foata, Norbert Sprenger, Florence Rochat, Sami Damak

**Affiliations:** Société des Produits Nestlé S.A., Nestlé Research, Route du Jorat, Vers-chez-les-Blanc, 1000, Lausanne 26, Switzerland

**Keywords:** Polysaccharides, Molecular biology

## Abstract

Numerous benefits of breastfeeding over infant formula are fully established. The superiority of human milk over bovine milk-based formula is partly due to human milk oligosaccharides (HMOs), a family of over 100 molecules present specifically and substantially in human milk that resemble mucosal glycans. To uncover novel physiological functions and pathways of HMOs, we screened a panel of 165 G-protein coupled receptors (GPCRs) using a blend of 6 HMOs (3′-O-sialyllactose (3′SL), 6′-O-sialyllactose (6′SL), lacto-N-tetraose (LNT), lacto-N-neo-tetraose (LNnT), 2-O-fucosyllactose (2′FL), and difucosyllactose (diFL)), and followed up positive hits with standard receptor assays. The HMO blend specifically activated GPR35. LNT and 6′SL individually activated GPR35, and they showed synergy when used together. In addition, in vitro fermentation of infant stool samples showed that 2′FL upregulates the production of the GPR35 agonist kynurenic acid (KYNA) by the microbiota. LNT + 6′SL and KYNA showed additive activation of GPR35. Activation by 6′SL and LNT of GPR35, a receptor mediating attenuation of pain and colitis, is to our knowledge the first demonstration of GPCR activation by any HMO. In addition, we demonstrated a remarkable cooperation between nutrition and microbiota towards activation of a host receptor highlighting the close interplay between environment and host-microbe interactions.

## Introduction

The benefits of breastfeeding over infant formula feeding are fully established^1^. The superiority of human milk over cow milk based infant formula may partly be due to the benefits of human milk oligosaccharides (HMOs), a family of over 100 molecules present in human milk in substantial amounts, making the third most abundant solid component of human milk, after lactose and fat^2^. The composition of oligosaccharides in milk varies widely between species^3^. Humans have the highest abundance and complexity of milk oligosaccharides of all studied species, although not every woman produces the full spectrum of oligosaccharides^4^. Farm animals, including cows have in their milk about 100th to 1000th the amount of oligosaccharides found in human milk, and fewer different molecules (reviewed in^2^).

HMOs are composed of a lactose backbone to which are attached N-acetylglucosamine, fucose or sialic acid, in various amounts and linkages. These vary from single additional molecule to large, more complex molecules^2^. The most abundant HMO in human milk is 2-O-fucosyl lactose (2′FL)^5,6^. HMOs are poorly absorbed^7,8^. HMOs are partly fermented in the colon by the intestinal microbiota and the rest is excreted in the stools either intact or in a modified form (reviewed by^9^).

HMOs are well known to serve as substrate for the beneficial microbiota of infants, thereby favoring the growth of “good” bacteria such as bifidobacteria^10–15^. In addition to protecting from pathogens by providing a competitive advantage to beneficial species, HMOs may also directly reduce microbial infections by acting as a decoy, preventing binding of infectious bacteria to the intestinal mucosa (reviewed by^16^), and by educating the immune system (reviewed by^17^). Clinical associations combined with basic research studies have suggested a role for HMOs in helping immune protection from infections^18^ and possibly also in helping prevent necrotizing enterocolitis^19,20^. Possibly mediated through a microbiota-gut-brain axis, HMOs are also shown in basic research models to improve cognition^21–23^. The immunomodulatory function of specific HMOs may be mediated through Toll like receptors^24^. Due to the high structural overlap between HMOs and glycans at the mucosal surface and cell glycocalix, where glycans exert modulatory roles, HMOs may also have such direct modulatory functions. Expectedly, this may be mediated through classical glycan binding proteins^25^, or yet to be discovered mechanisms.

G protein-coupled receptors (GPCRs) constitute the largest family of receptors in several organisms including human^26^ and are implicated in almost every physiological function such as development, taste, olfaction, regulation of heart rate, hormone signaling, neurotransmission and many others^27^. They respond to molecules with a large variety of structures and sizes, ranging from protons^28^ to proteins^29^. Many GPCRs are expressed in the gastrointestinal tract^30^ and are therefore accessible targets for HMOs. We reasoned that some physiological functions of HMOs could be mediated by GPCRs and could be discovered by identifying GPCRs that respond to HMOs. For this purpose, we screened a panel of 165 GPCRs using a blend of 6 manufactured HMOs.

## Results

### Screen of the GPCR panel with a 6-HMO blend

**Figure 1:**
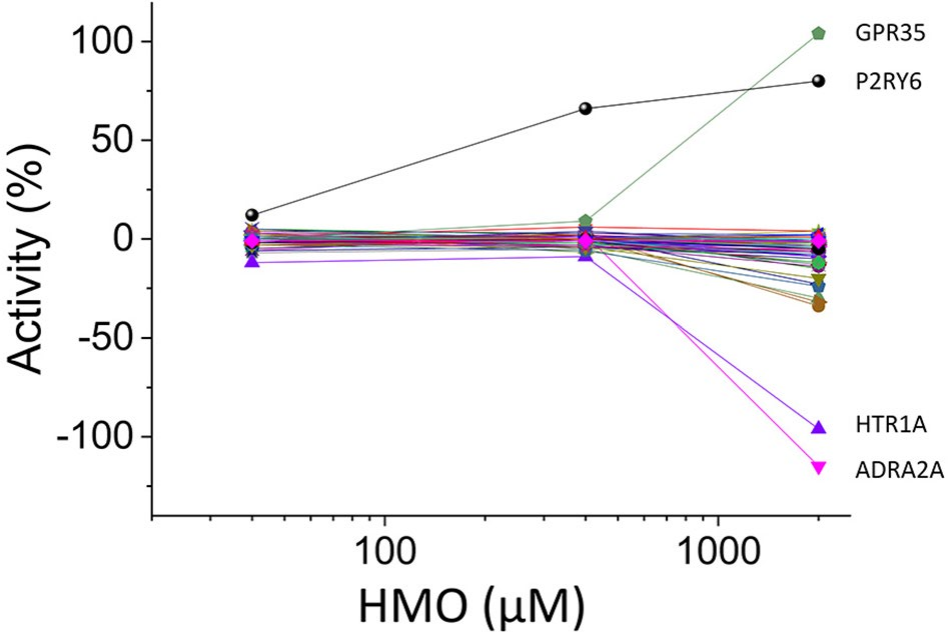
GPRCR panel screen with HMOs. Responses in agonist mode to a 6-HMO blend (3′SL, 6′SL, LNT, LNnT, 2′FL, diFL) of 165 cell lines each expressing a specific GPCR. Datapoints are means of technical duplicates. GPR35 and P2RY6 show activation and HTR1A and ADRA2A show inactivation with 2 mM HMOs. Additional controls showed that the apparent activation of P2RY6 was caused by a contaminant.

An equimolar blend made of 3′-O-sialyllactose (3′SL), 6′-O-sialyllactose (6′SL), lacto-N-tetraose (LNT), lacto-N-neo-tetraose (LNnT), 2-O-fucosyllactose (2′FL), and difucosyllactose (diFL) was used to screen a panel of 165 GPCRs at concentrations of 40 μM, 400 μM and 2 mM each. In agonist mode, the blend activated GPR35 at 2 mM (Fig. 1). None of the other 164 receptors showed activation with the HMO blend except for P2RY6 for which additional control experiments using hydrolysed agonists showed that activation was caused by a contaminant (Supplementary Figure S1). A small number of receptors were inhibited by the HMO blend at 2 mM. Presumably, those receptors have a constitutive activity that is antagonized by one or more HMOs from the blend. Of those, 5-hydroxytryptamine receptor 1A (HTR1A) and adrenoceptor alpha 2A (ADRA2A) showed ~ 100% inhibition (Fig. 1). Additional dose–response experiments in antagonist mode did not confirm with certainty the specificity of those inhibitory responses (not shown), which were therefore not further explored.

### Activation of GPR35 by individual HMOs

To confirm the results of the HMO blend and to determine which individual HMOs activate GPR35, dose response curves were obtained from the GPR35 receptor with each of the HMOs used in the blend individually.

**Figure 2:**
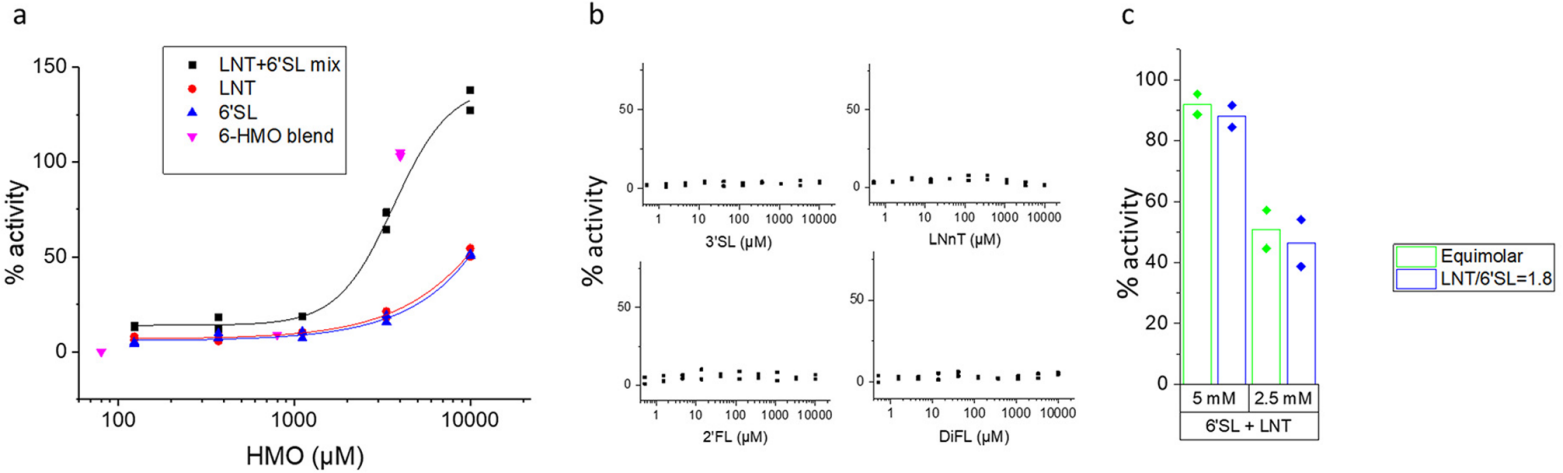
Activation of GPR35 by HMOs. (**a**) Dose response of GPR35 activation in arrestin mode by 6′SL, LNT and equimolar 6′SL + LNT. The concentrations indicated are total concentrations of HMOs. The responses of the three concentrations of 6 HMOs (from Fig. 1) are indicated. (**b**) Dose response of GPR35 stimulation in arrestin mode by 3′SL, LNnT, 2′FL and DiFL showing no activation of the receptor by any of those HMOs. (**c**) Responses of GPR35 in arrestin mode to equimolar LNT and 6′SL or to LNT and 6′SL at a ratio of 1.8, showing no difference between ratios of 1:1 and 1.8:1. The concentrations shown are total concentration of HMOs.

**Figure 3:**
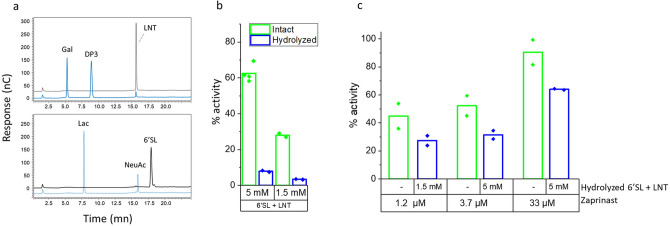
Specificity of the GPR35 response to LNT and 6′SL. (**a**) HPAEC/PAD analysis of 6′SL and LNT before (black trace) and after (blue trace) treatment with Neuraminidase (6′SL) or β-galactosidase (LNT) showing complete digestion of the 6′SL and near complete digestion of LNT. The identities of the peaks are indicated. Gal, Galactose; DP3, agalacto-LNT; LNT, Lacto-N-tetraose; 6′SL, 6′Sialyllactose; NeuAc, N-Acetylneuraminic acid. nC: nanocoulomb. (**b**) Response of GPR35 to digested and undigested mix of LNT and 6′SL showing a response with the undigested mix and background response to the digested mix, thereby demonstrating the specificity of the GPR35 response. (**c**) Response of GPR35 to its agonist Zaprinast plus digested HMOs, showing only partial decrease of the GPR35 response to Zaprinast by the HMO digestion product.

GPR35 was activated by LNT and by 6′SL at concentrations ≥ 3.3 mM and by an equimolar mix of LNT and 6′SL at concentrations ≥ 1.66 mM each with EC50 = 2.18 mM (Fig. 2) but not by any of the other 4 HMOs from the 6-HMO blend. The response to 1.66 mM LNT + 1.66 mM 6′SL (69%) is greater than the response to 3.3 mM LNT (17%) or the response to 3.3 mM 6′SL (20%) indicating synergy between the two HMOs. Changing the ratio of LNT/6′SL from equimolar to the more physiological ratio of 1.8 did not affect the magnitude of the response (Fig. 2C). Hydrolysis of 6′SL and LNT with neuraminidase and β-galactosidase, respectively, eliminated the response (Fig. 3). The 6′SL and LNT hydrolysis product had only a minor inhibitory effect on activation of GPR35 by the synthetic agonist Zaprinast (Fig. 3C), indicating that the lack of activation of GPR35 by hydrolysed HMOs is indeed due to elimination of the agonist, and not to nonspecific inhibition of the receptor assay by the hydrolysis products. These data demonstrate that activation of GPR35 is indeed caused by the 6′SL and LNT molecules and not by a contaminant.

### Additive effect of 6′SL + LNT and kynurenic acid on GPR35

**Figure 4:**
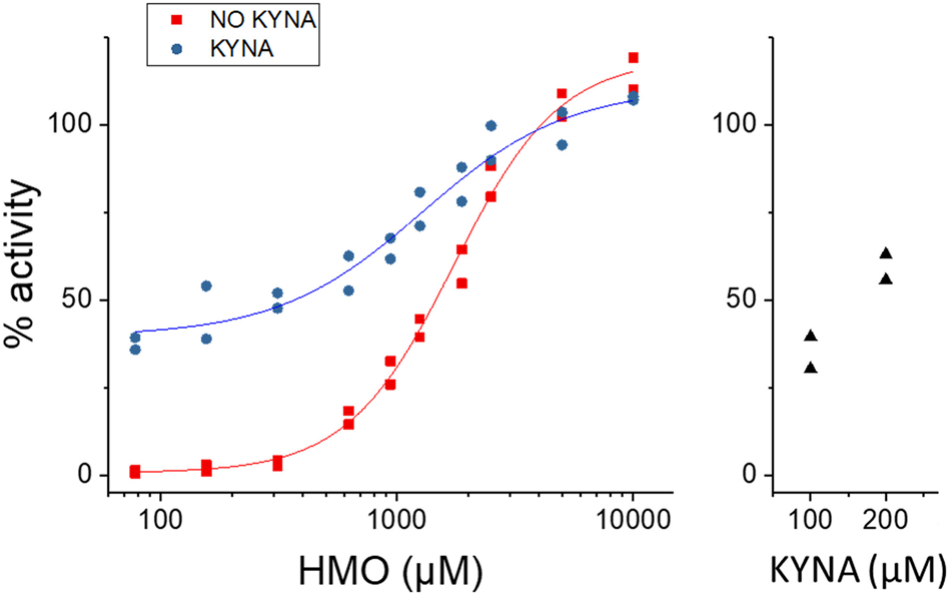
Additive effect of HMOs and KYNA on GPR35 activation. Dose response of GPR35 activation in arrestin mode to equimolar 6′SL + LNT with or without 200 µM KYNA, and to 100 µM and 200 µM KYNA, showing additive effect at concentrations between 940 µM and 2500 µM. This experiment was conducted three times, with comparable results.

Kynurenic acid (KYNA) is a natural partial agonist of GPR35^31^, which is present in the intestinal lumen. Stimulation of GPR35 with 6′SL and LNT plus 200 µM KYNA showed an additive effect at HMO concentrations between 940 µM and 2500 µM (Fig. 4).

### The HMO 2′FL increases the amount of KYNA produced by the microbiota

**Figure 5:**
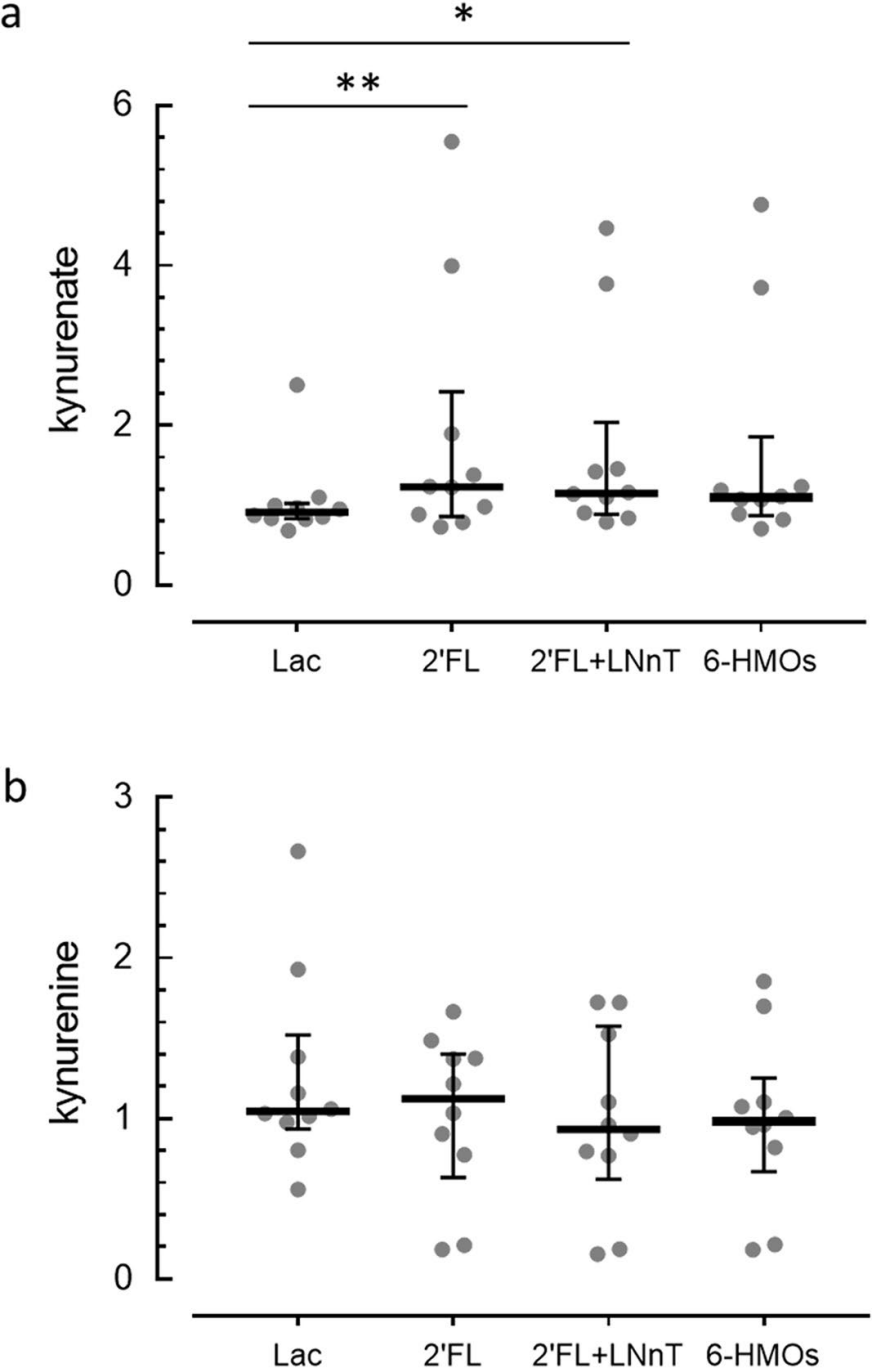
In vitro fermentation of stool samples in the presence of HMOs. Concentration in arbitrary units of kynurenate (KYNA) (**a**) and kynurenine (**b**) in the supernatant of infant stool samples fermented in vitro for 48 h in the presence of lactose, 2′FL, 2′FL + LNnT or 6-HMO blend. The concentration of KYNA but not kynurenine is increased in the presence of the HMOs, compared to lactose. Individual data with median ± interquartile ranges are shown. *p < 0.05. **p < 0.01. N = 10 different donors.

**Figure 6:**
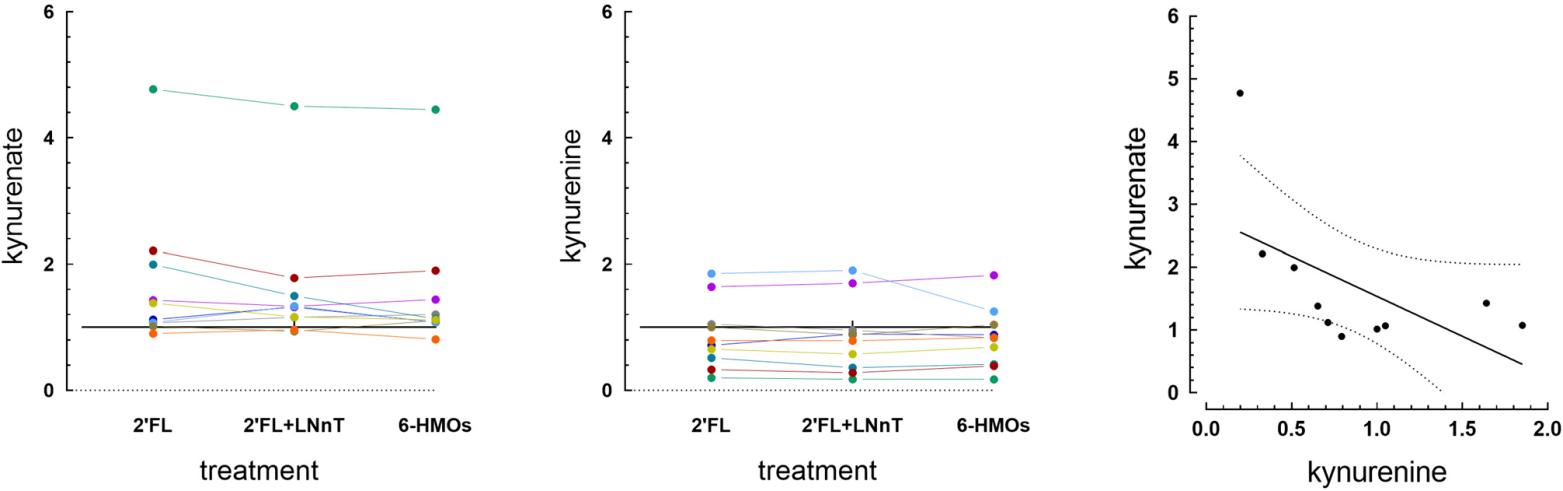
Relationship between kynurenine and KYNA in fermented stool samples. (**a**) Ratios of kynurenate (KYNA) in the supernatant of infant stool samples fermented in vitro for 48 h in the presence of 2′FL, 2′FL + LNnT or 6HMO to KYNA fermented in the presence of lactose. (**b**) Same ratios as in (**a**) for kynurenine. (**c**) Linear correlation between the ratios for KYNA and kynurenine for fermentation in presence of 2′FL. Each datapoint is an individual donor, dots with the same color represent samples from the same donor. N = 10 donors. The horizontal line in (**a**) and (**b**) indicates a ratio of 1, i.e. no difference between HMO and lactose.

We reasoned that the physiological benefits of HMOs through GPR35 might be mediated by different pathways, one being direct activation of the receptor and the second being through KYNA. To test this hypothesis, we mined an existing metabolomics dataset to assess the effect of supplementing the media of in vitro fermented infant stool samples with various combinations of HMOs on the relative concentration of KYNA in the media. We found that compared to lactose, 2′FL, or a combination of 2′FL and LNnT significantly increased KYNA by 1.76 X and 1.61 X (*p* = 0.01 and *p* = 0.046, respectively) in the fermentation supernatant (Fig. 5A). The 6-HMO blend resulted in a trend for increased KYNA (1.56 X mean increase compared to lactose, *p* = 0.07). There is no significant difference between any of the HMO treatments suggesting that the increase of KYNA is mostly due to 2′FL. In contrast to KYNA, its precursor kynurenine was not significantly affected by any of the HMO combinations, compared to lactose (*p* = 0.23, Fig. 5B). When we examined the ratios treatment/lactose for KYNA and kynurenine, we found a trend for a negative correlation (R^2^ = 0.34, *p* = 0.077 for 2′FL), with KYNA increasing with the treatments and kynurenine tending to decrease (Fig. 6). Thus, it appears that addition of 2′FL modulates primarily the last step of the pathway that produces KYNA from tryptophan, in which the enzymes kynurenine aminotransferases 1 and 2 convert kynurenine into KYNA.

## Discussion

HMOs support several health benefits by feeding and favoring the growth of beneficial microbes, and by acting as a decoy to prevent pathogens from binding to the intestinal mucosa. Whereas some target molecules mediating HMO activity such as Toll like receptors and Dendritic Cell-Specific Intercellular adhesion molecule-3-Grabbing Non-integrin (DC-SIGN) have been proposed^24,25^, we demonstrate here for the first time the existence of another mechanism of action of HMOs, through activation of GPCRs.

We screened a panel of 165 GPCRs with a mixture of six HMOs. Those were selected based on abundance in milk and commercial availability. Only GPR35 out of the 165 GPCRs tested showed specific activation. Multiple and stringent controls are required when working with HMOs in in vitro tests, as these molecules have low potency, being active in the high micromolar to millimolar range. At those concentrations, a potent contaminant could have an impact even if it is present at below threshold detection level. Importantly, hydolysing 6′SL and LNT resulted in loss of activation of GPR35, indicating that it was specific action of 6′SL and LNT and not a contaminant that was responsible for GPR35 activation.

GPR35 is expressed in several organs including lung, stomach, small intestine, colon, spleen, skeletal muscle, dorsal root ganglion, uterus, spinal cord, heart, liver, bladder, brain, mast cells, and basophils^32^. The highest levels of expression are in the gastrointestinal tract and the spleen^31^, and expression in the intestinal surface epithelium has been documented in a number of studies by in situ hybridization^31^, immunohistochemistry^33^, and RNA microarray analysis of sorted intestinal epithelial cells^34^.

The EC50s for activation of GPR35 by 6′SL + LNT is 2.18 mM each. The concentrations of HMOs in mature milk is variable with time, geographic location, activity of the Fut2 gene (secretor status), and between individuals. Ranges between 2.2 and 0.95 mM for LNT and between 0.89  and 0.2 mM for 6′SL have been reported^5,6^. As milk is ingested and progresses through the infant gastrointestinal tract, water gets absorbed and the concentration of the HMOs may increase, thereby possibly reaching a concentration at which it can activate GPR35. In addition, because of the additive effect of KYNA on GPR35 activation, even lower concentrations of 6′SL and LNT may be physiologically active. GPR35 is expressed in intestinal epithelial cells and therefore accessible to ingested HMOs. Thus, it is reasonable to expect a physiological effect of those oligosaccharides through activation of gastrointestinal tract-expressed GPR35, based on their concentrations in milk. Physiological effects of the tested HMOs through GPR35 in organs outside the gastrointestinal tract and the mouth are unlikely. Only ~ 1% of HMOs are absorbed, and are diluted in the extracellular milieu following absorption. Therefore, the concentrations that reach organs other than the gut are likely too low to activate GPR35 expressed in those organs.

GPR35 has been proposed to be part of several physiological functions (for review see^32^). Here we will focus on those related to its expression in the gastrointestinal tract as they are the functions that can potentially be modulated by HMOs through GPR35.

Many lines of evidence indicate that GPR35 may have a role in attenuating pain. In mice, systemic pre-treatment with the KYNA precursor L-kynurenine or the GPR35 agonist Zaprinast significantly reduced the number of writhes caused by acetic acid i.p. injection^35^. In a rat model of neuropathic pain, intrathecal administration of Zaprinast diminished pain symptoms and increased the effectiveness of opioids^36^. IP injection of Zaprinast or KYNA in rats also attenuates PGE2-induced thermal hyperalgesia^37^. In all cases, the effect of the agonists on pain was blocked by GPR35 antagonists. Mucosal KYNA is decreased in patients with irritable bowel syndrome (IBS), a condition characterised by abdominal pain and transit disorders^38^. Whether intestinal epithelium-expressed GPR35 also plays a role in attenuating abdominal pain is unknown. Importantly, GPR35 is expressed in enterochromaffin cells^34^, cells that produce serotonin, an important mediator of abdominal pain^39^. It is tempting to speculate that 6′SL, LNT and KYNA may play a role in attenuation of abdominal pain in infants at least in part by modulating the production or secretion of serotonin by enterochromaffin cells through activation of GPR35.

Two human genome wide association studies (GWAS) have found associations between single nucleotide polymorphisms (SNPs) in GPR35 and ulcerative colitis^40,41^. Studies investigating the role of GPR35 in dextran sodium sulfate (DSS)-induced colitis in mice, one using agonists and antagonists of GPR35^33^ and the other GPR35 knockout mice^42^, concluded that GPR35 could play a part in protecting mice from colitis. Experiments using agonists and antagonists in vitro indicated that activation of GPR35 stimulates epithelial cell migration^33^, the first step in wound healing. Another study showed that GPR35 promotes proliferation of intestinal epithelial cells^43^. In newborn rats, supplementation of the dam with the GPR35 agonist KYNA leads to increased KYNA in the milk of the nursing dams and to increased intestinal absorptive surface, mucosa thickness and increased number of mitosis in the intestinal crypt of nursing pups^44^. A study using GPR35 agonist KYNA suggested that GPR35 may be involved in reducing inflammation^45^. Together these data suggest that GPR35 may play a role in attenuating colitis, and that this effect could be due to its role in promoting wound healing and in attenuating inflammation.

**Figure 7:**
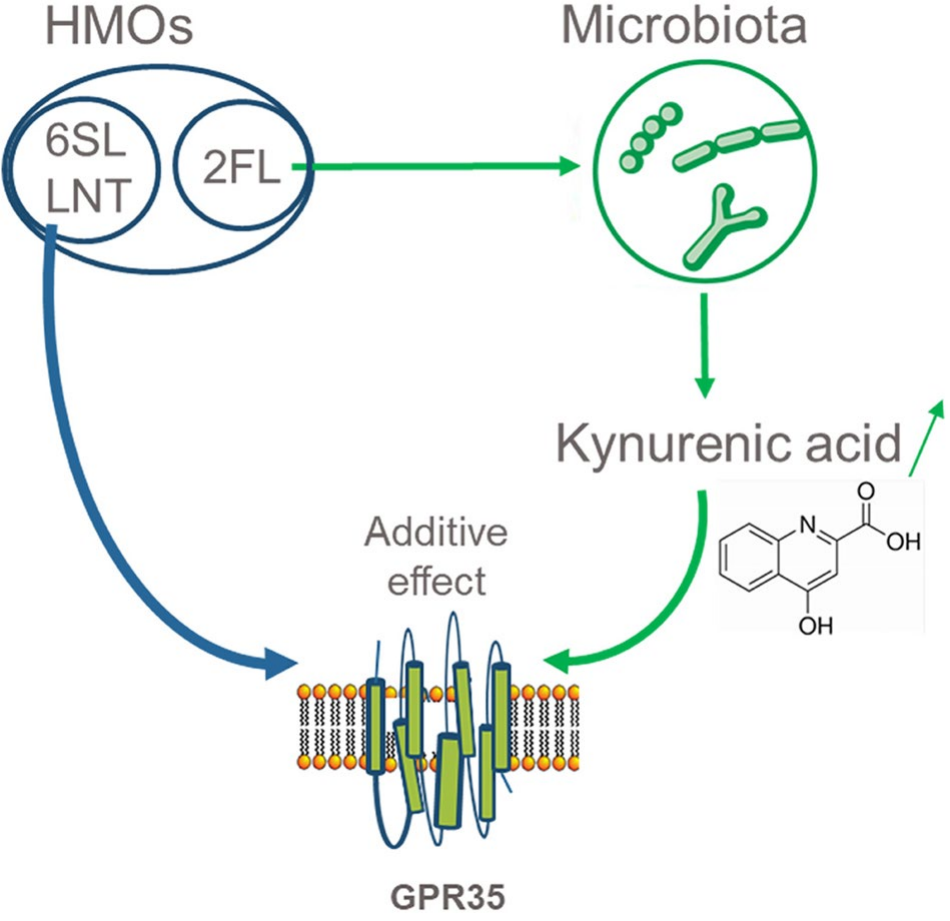
Model of GPR35 activation through different pathways. One pathway is direct activation by 6′SL and LNT, the second is increased production of KYNA by the microbiota which in turn activates GPR35.

A number of endogenous molecules have been shown to activate GPR35 with modest potency and in some cases without strict specificity^46^. Kynurenic acid, a metabolite of tryptophan is often used as typical agonist of GPR35^31^. Here we showed that 6′SL + LNT and KYNA have additive effects on activation of GPR35. Moreover, we showed that 2′FL increases KYNA concentrations in in vitro fermentation of infant stools. Therefore, HMOs appear to have at least two pathways to activate GPR35. One is through direct interaction by 6′SL and LNT and the other by 2′FL through the gut microbiota increasing the concentration of KYNA (Fig. 7). The indirect pathway may constitute one of the mechanisms by which 2′FL reduces colitis in pre-clinical models^19,20,47^. Whether these findings at the receptor level and in pre-clinical models will translate into clinical benefits will have to be determined in future studies.

In addition to this remarkable cooperation between nutrition and microbiota towards activation of a receptor, we showed for the first time that HMOs are able to activate a GPCR, a receptor family hitherto not known to be modulated by HMOs. Here, we have tested only six out of the over 100 HMOs present in milk, and only on a panel including most deorphanized non olfactory receptors, but not the orphan receptors. We believe the large number of HMOs together with the large number of microbial metabolites in the gut ecosystem constitute a poorly explored library of natural compounds with a large potential to impact many physiological functions. To this end, a receptor screen as used here is a powerful discovery tool as a first step to generate testable hypotheses in clinical settings.

## Methods

All methods were carried out in accordance with relevant guidelines and regulations.

### Screen of a GPCR panel

#### Principle of the test

The screen was conducted by DiscoverX (Fremont, Ca, USA) using the PathHunter β-Arrestin assay as described (https://www.discoverx.com/arrestin). This assay monitors the activation of a GPCR expressed in cultured cells, using Enzyme Fragment Complementation (EFC), with β-galactosidase (β-Gal) as the functional reporter. The enzyme is split into two inactive complementary portions, EA and PK, expressed as fusion proteins in the cell. EA is fused to β-Arrestin and PK is fused to the GPCR of interest. When the GPCR is activated and β‐Arrestin is recruited to the receptor, PK and EA complementation occurs, restoring β‐Gal activity which is measured using Chemiluminescent Detection Reagents. The cell lines used in this study have CHO‐K1 or U2OS backgrounds.

#### Receptors and compounds

The GPCR gpcrMAX panel, which contains 165 deorphanized human receptors, was screened with an equimolar mixture of the synthetic HMOs 3′-O-sialyllactose (3′SL), 6′-O-sialyllactose (6′SL), lacto-N-tetraose (LNT), lacto-N-neo-tetraose (LNnT), 2-O-fucosyllactose (2′FL), and difucosyllactose (diFL) tested at 40 μM, 200 μM and 2 mM each. All HMOs were obtained from Glycom A/S, Denmark.

#### Receptor assay

Receptor assay was carried out as described^48^. PathHunter cell lines were expanded from frozen stocks according to standard procedures. Five thousand cells were seeded in a total volume of 20 μL into each well of white walled, 384‐well microplates and incubated at 37 °C for the appropriate time prior to testing. All testing was done in duplicate.

On each test day, a 5 × compound working intermediate was prepared in phosphate buffered saline (PBS) for each test concentration. Five µL of 5 × sample was added to cells and incubated at 37 °C or room temperature for 90 or 180 min.

Assay signal was generated through a single addition of 12.5 or 15 μL (50% v/v) of PathHunter Detection reagent cocktail (DiscoverX), followed by a one hour incubation at room temperature. Microplates were read following signal generation with a PerkinElmer Envision instrument for chemiluminescent signal detection.

Compound activity was analyzed using CBIS data analysis suite (ChemInnovation, CA). Percentage activity was calculated using the following formula:

$${\%} \,\mathrm{Activity} = 100{\% }\times (\mathrm{mean \,RLU \,of \,test \,sample}-\mathrm{ mean \,RLU \,of \,vehicle \,control}) / (\mathrm{mean \,MAX \,control \,ligand}-\mathrm{ mean \,RLU \,of \,vehicle \,control}).$$

Receptor assays were conducted in duplicate.

### Enzymatic hydrolysis of HMOs

#### Hydrolysis of LNT by β-galactosidase

LNT was treated with β-galactosidase purified from aspergillus Niger (Megazyme, ref: E-AGLAN) using the following protocol: in a 1.5 ml screw cap tube with external thread, we mixed 84 mg of LNT in 900 µl of 100 mM acetate buffer (pH 4.5) and 100 µl of enzyme (400 U). The mix was incubated for 72 h at 55 °C in a ThermoMix shaker with 450 rpm shaking. The inactivation of the enzyme was done by heat treatment of 5 min at 100 °C. The sample was stored at − 20 °C until further analysis.

#### Hydrolysis of 6′SL by neuraminidase

6′SL was treated with neuraminidase purified from *Clostridium perfringens* (Megazyme, ref: E-SIALCP) using the following protocol: in a 1.5 ml screw cap tube with external thread, we mixed 78 mg of 6′SL in 900 µl of 50 mM phosphate buffer (pH 6.0) and 50 µl of enzyme (50 U). This mix was incubated for 72 h at 37 °C in a ThermoMix shaker with 450 rpm shaking. The inactivation of the enzyme was done by heat treatment for 5 min at 100 °C. The sample was stored at − 20 °C until further analysis.

#### Control of the hydrolysis

We confirmed that the digestions of LNT and 6′SL were complete by quantifying them together with their hydrolysis products, Galactose, Lacto-N-triose, N-acetyl-neuraminic acid and lactose using high performance anion exchange chromatography (HPAEC) with a CarboPac PA1 analytical column coupled to a pulsed amperometry detector (ICS3000, Thermo Fischer Dionex, Sunnyvale, USA) essentially as previously described^49^.

### Relative abundance of kynurenic acid in fermented stool samples

We mined an unpublished metabolomics dataset to determine the effect of HMO fermentation by the intestinal microbiota on the production of kynurenic acid.

#### Culture of stool samples

All parents gave consent to have their child’s stools deposited into a biobank for further analysis. The protocol has been approved by the cantonal ethics committee (Commission cantonale (VD) d'éthique de la recherche sur l'être humain) . Freshly passed stool samples from ten 3–4 month-old infants, seven breast-fed and three formula-fed were used to inoculate YCFA medium lacking carbohydrates (CHO-free). The formulae were standard commercial products selected by the parents. YCFA is a complex medium containing tryptone, yeast extract, and various minerals, vitamins, and short chain fatty acids; however, the typical CHO components (glucose, maltose, and cellobiose) were excluded in this case. The study design involved supplementing the CHO-free YCFA medium with different carbohydrates 0.5% final concentration, either lactose (control) or 3 test conditions: 2′FL alone, a combination of 2′FL and LNnT (2:1, w/w), or a combination of 2′FL, diFL, 3′SL, 6′SL, LNT and LNnT (55:6:7:9:18:5) to mimic the average ratio in human milk. Fermentations were carried out in 4 parallel batch fermenters under anaerobic condition. Four fermentation conditions were conducted for each subject, and supernatants were collected after 24 h and 48 h at 37 °C. Collected supernatant samples were stored at − 80 °C.

#### Metabolomics

Analysis was done by Metabolon (Morrisville, North Carolina, USA) using their HD4 Platform. Samples were extracted and split into equal parts for analysis on LC/MS/MS and Polar LC platforms. Proprietary software was used to match ions to an in-house library of standards for metabolite identification and for metabolite quantitation by peak area integration.

The statistical analyses were performed on natural log-transformed data. Treatment effect was evaluated using a non-parametric paired group test (Friedman’s test; GraphPad Prism, version 7.01). Dunn's multiple comparisons test was used to determine which treatment differed from lactose.

## Supplementary information

Supplementary Information.

## Data Availability

The detailed results of the receptor panel screen are available upon request from the corresponding author.
