# Supplementary material for: Activation of the G-protein coupled receptor GPR35 by human milk oligosaccharides through different pathways
Source: Sci Rep. 2020 Sep 30;10:16117. doi: 10.1038/s41598-020-73008-0 (PMC7528069; doi:10.1038/s41598-020-73008-0)
Supplement: Supplementary file 1 — Supplementary Information. [file 41598_2020_73008_MOESM1_ESM.pdf]

Activation of the G-protein coupled receptor GPR35 by human milk oligosaccharides through different pathways

Francis Foata, Norbert Sprenger, Florence Rochat, Sami Damak*

Supplementary information

P2RY6 was activated by 3’SL (supplementary Figure S1A) but not by any of the remaining 5 HMOs from the blend. To examine the possibility that activation of P2RY6 by 3’SL was caused by a contaminant in the 3’SL sample, we treated the sample with neuraminidase to specifically digest 3’SL and verified by High Performance Anion Exchange Chromatography equipped with pulse amperometric detection (HPAEC-PAD) that the digestion was complete. The response of P2RY6 to the digested 3’SL was slightly diminished compared to that of intact 3’SL but a substantial response remained (supplementary Figure S1B). These data suggested that the P2RY6 response was caused by a contaminant. Since the natural agonist of P2RY6 is uridine di phosphate (UDP), we treated the 3’SL sample with apyrase, an enzyme that digests nucleotides di- and tri-phosphate. Apyrase-treated 3’SL did not activate P2RY6, confirming that the observed response with the intact sample is caused by a contaminant (Figure S1C).

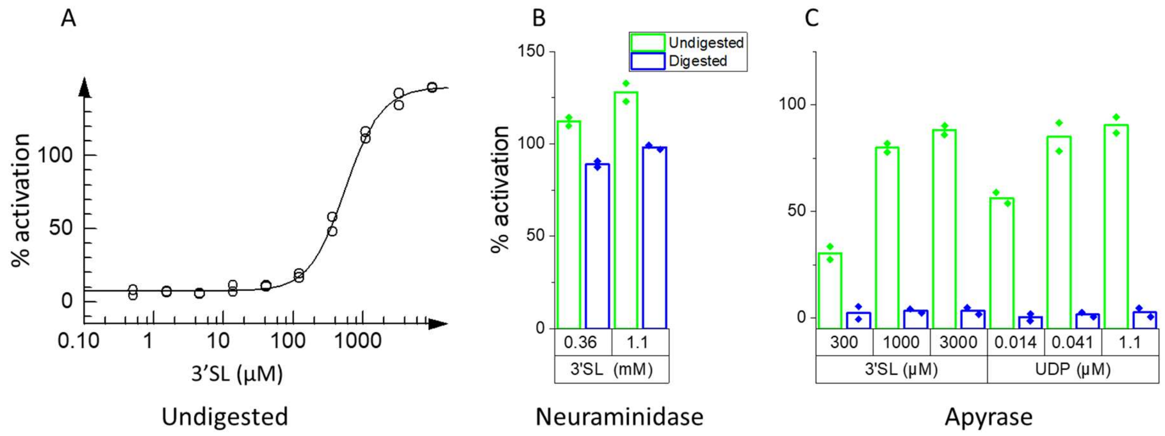

Figure legend

Supplementary Figure S1. Response of P2RY6 to digested and undigested mix of 3’SL showing a response with the undigested 3’SL and neuraminidase-treated 3’SL and background response to apyrase-treated 3’SL.
